# Use of the "ee" Maneuver in a Patient With Dysphagia Due to Severe Pseudobulbar Palsy

**DOI:** 10.7759/cureus.30164

**Published:** 2022-10-11

**Authors:** Kenjiro Kunieda, Tomohisa Ohno, Kazuo Tanahashi, Takashi Shigematsu, Ichiro Fujishima

**Affiliations:** 1 Neurology, Gifu University Graduate School of Medicine, Gifu, JPN; 2 Rehabilitation Medicine, Hamamatsu City Rehabilitation Hospital, Hamamatsu, JPN; 3 Dentistry, Hamamatsu City Rehabilitation Hospital, Hamamatsu, JPN

**Keywords:** stroke, maneuver, deglutition, oral phase, tongue, rehabilitation, pseudobulbar palsy, swallow, cognitive impairment, dysphagia

## Abstract

Dysphagia in pseudobulbar palsy is characterized by impairment of the oral stage of swallowing. The flow of the bolus from the oral cavity into the pharynx at the fauces may be blocked in some patients, which prevents the bolus flow by contact of the tongue with the palate. Herein, we demonstrated a case with pseudobulbar palsy who could deliver bolus from the oral cavity to the pharynx by vocalizing "ee." An 81-year-old man presented with a recurrent cerebral infarction due to cardiogenic embolism. He presented with pseudobulbar palsy and had severe dysphagia due to bilateral cerebral hemisphere lesions. On day 84, a videofluoroscopic examination of swallowing was performed in a 30° reclining posture. When the bolus reached the posterior part of the tongue in the oral cavity, the clinician asked the patient to say "ee." The base of the tongue moved forward and downward, and the anterior to the middle part of the tongue was elevated in the mouth. As a result, the fauces opened, the functional blockage was released, and the bolus flowed into the pharyngeal cavity. Shortly after the swallowing reflex, the bolus passed through the pharynx. We have named this swallowing maneuver the "ee" maneuver. The "ee" maneuver can be one of the swallowing methods to improve bolus transport from the oral cavity to the pharynx in patients with dysphagia and cognitive impairment due to pseudobulbar palsy.

## Introduction

Swallowing is divided into the oral preparatory stage, the oral stage, the pharyngeal stage, and the esophageal stage. The oral preparatory stage includes masticating, that is, mixing the food with saliva. In the oral stage, the tongue contacts the hard palate and delivers the bolus into the pharynx after the formation of the bolus.

Pseudobulbar palsy occurs due to bilateral cortico-bulbar tract impairment [1], and it is characterized by dysarthria, dysphagia, facial and tongue weakness, emotional lability, and cognitive impairment. Dysphagia due to pseudobulbar palsy is common and characterized by impairment of the oral stage of swallowing [2,3]. In these patients, oral transit time is prolonged due to poor bolus transport from the oral cavity to the pharynx. Furthermore, in some patients, the bolus may be functionally blocked at the fauces due to contact of the tongue with the palate and can be prevented from flowing from the oral cavity into the pharynx. The central pattern generator (CPG) for swallowing, located in the medulla, controls the oropharyngeal stage of the swallowing sequence [4]. The CPG is not impaired, and pharyngeal swallowing function is preserved in patients with pseudobulbar palsy [5]. Unlike the oral stage, the pharyngeal stage is an involuntary process. As the food bolus reaches the pharynx, the swallowing reflex is triggered. Therefore, delivering the bolus smoothly into the pharynx is important for swallowing rehabilitation in dysphagia patients due to pseudobulbar palsy.

The aim of this report was to demonstrate a patient with dysphagia due to pseudobulbar palsy who delivered the bolus from the oral cavity to the pharynx by vocalizing "ee."

## Case presentation

**Figure 1:**
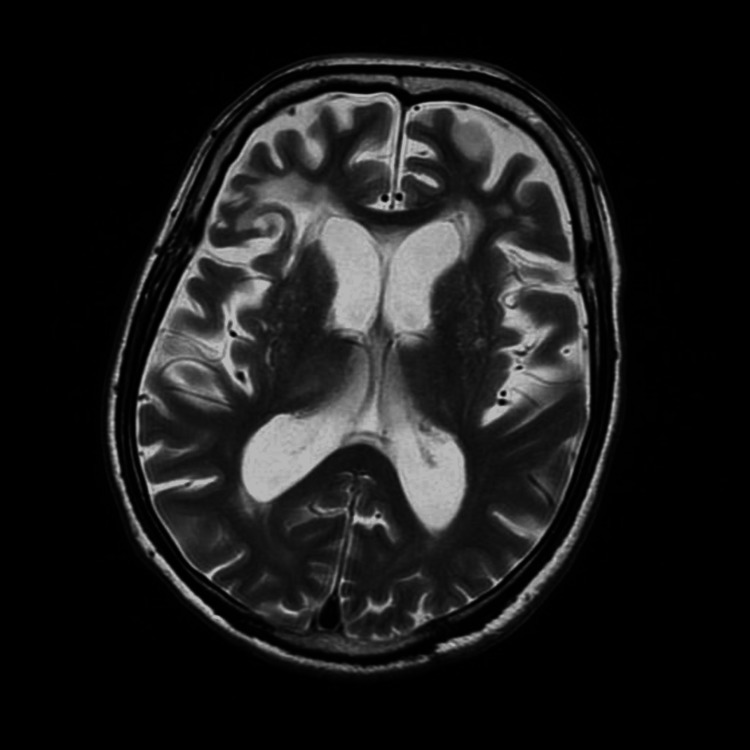
T2-weighted image shows cerebral infarctions of the bilateral cerebral hemispheres

An 81-year-old man presented with right-sided hemiplegia and dysarthria. He was diagnosed with a recurrent cerebral infarction due to cardiogenic embolism because his medical history included a cardiac source of embolism due to atrial fibrillation at 77 years old. Anticoagulation therapy was continued. He also presented with pseudobulbar palsy and had severe dysphagia owing to bilateral cerebral hemisphere lesions (Figure 1). He was fed by tube. He underwent medical treatment and physical and swallowing rehabilitation at an acute hospital.

Fifty-two days after the onset, the patient was transferred to the rehabilitation hospital, and at the time of transfer, he needed a wheelchair and assistance for personal care. He showed severe right-sided hemiplegia. His body mass index was 19.8 kg/m2, with no obvious loss of muscle mass suggesting sarcopenia. He had severe cognitive impairment and little speech. Cognitive assessments, such as the Mini-Mental State Examination (MMSE), were not performed because he had difficulty answering the MMSE questionnaires adequately. He was only able to follow very simple instructions. Although the assessment of cranial nerve palsy was limited due to cognitive dysfunction, a videoendoscopic examination of swallowing revealed no paralysis of the pharynx and vocal cords. On day 59, an initial videofluoroscopic examination (VF) of swallowing was performed in a reclining posture. A small amount of jelly and thickened liquid was used to evaluate swallowing function. The bolus transport from the oral cavity to the pharynx was poor and occasionally blocked by the posterior part of the tongue. The swallowing reflex was delayed, and the pharyngeal residue was observed. After this VF, swallowing training was carefully carried out using a small quantity of food, such as a spoonful of jelly, paste, and thickened liquid, in the reclining posture. The amount of food intake was increased gradually, but bolus transport from the oral cavity to the pharynx had worsened. He developed aspiration pneumonia and was treated with antibiotics. The patient exhibited severe dysphagia classified as level 3 via the Food Intake LEVEL Scale (FILS), a 10-point observer rating scale measuring the severity of dysphagia (swallowing training using a small quantity of food is performed) [6].

The "ee" Maneuver

**Figure 2:**
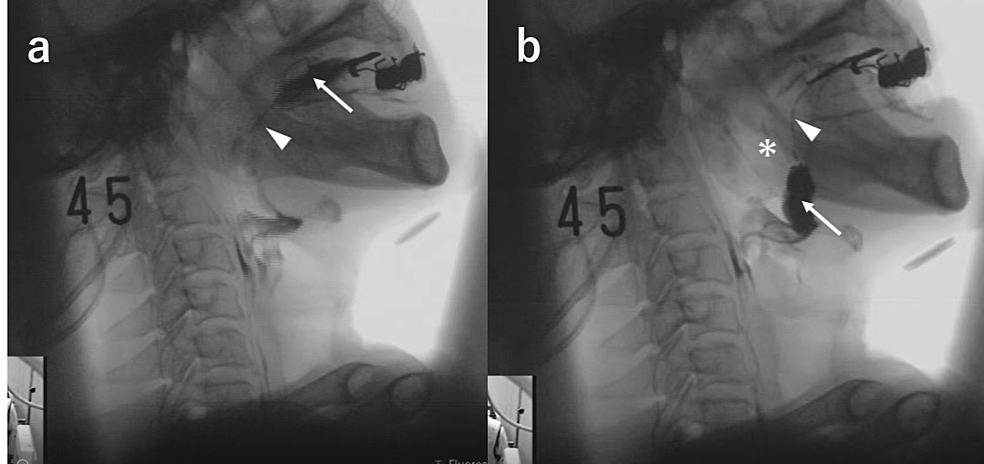
a) Blockage at the fauces due to contact of the anterior to the middle part of the tongue with the palate, b) Performing the “ee” maneuver When the patient was instructed to say "ee", the base of the tongue moved forward and downward (arrowhead), and the anterior to the middle part of the tongue was elevated at a higher position in the mouth. As a result, the fauces opened (asterisk). Immediately after that, the bolus flowed from the oral cavity into the pharyngeal cavity (arrow).

Follow-up VF at 84 days after onset revealed that the bolus did not flow from the oral cavity into the pharynx. The bolus was retained in the oral cavity because of being blocked functionally at the fauces due to the posterior part of the tongue pressing against the palate (Figure 2). The bolus remained in the oral cavity even when the physician instructed the patient to swallow. When the bolus reached the posterior part of the tongue in the oral cavity in the reclining position in the lateral view of VF, the clinician asked the patient to say "ee." The base of the tongue moved forward and downward, and the anterior to the middle part of the tongue was elevated in the mouth. Furthermore, the soft palate was elevated. As a result, the fauces opened, the functional blockage was released, and the bolus flowed into the pharyngeal cavity (Video 1). Performing the "ee" maneuver improved bolus blockage and allowed movement from the oral cavity to the pharynx by moving the base of the tongue forward and downward. Shortly thereafter, the bolus flowed into the pharynx (Figure 2). Initiation of the pharyngeal swallow was slightly delayed, but once the swallowing reflex was triggered, the bolus passed through the pharynx into the upper esophagus without aspiration. This technique was also used in conjunction with swallowing training (e.g., breathing training, neuromuscular electrical stimulation, and direct therapy with reclining posture) by a speech therapist. The bolus flowed into the pharynx during the "ee" vocalization. The amount of swallowing-adjusted food intake gradually increased. Ultimately, the patient's dysphagia improved to FILS level 7 (easy-to-swallow food is orally ingested in three meals; no alternative nutrition is given). On day 132 after the onset, he was discharged to a nursing home. A wheelchair was necessary at discharge. Cognitive dysfunction and right-sided paralysis remained, and the patient required nursing care.

**Video 1:** Performing the “ee” maneuver When the patient was instructed to say "ee," the base of the tongue moved forward and downward, and the tongue was elevated at a higher position in the mouth. As a result, the fauces opened. Immediately after that, the bolus flowed from the oral cavity into the pharyngeal cavity.

## Discussion

To our knowledge, this is the first report describing a unique swallowing technique to improve bolus transport from the oral cavity to the pharynx by producing the sound "ee" in a patient with pseudobulbar palsy. The authors have named this swallowing method the "ee" maneuver.

In our case, when the patient was asked to perform the "ee" maneuver, the fauces opened. As a result, the blockage was released, and the bolus flowed into the pharyngeal cavity. Producing the sounds "ah" or "oh" also opens up the fauces; however, production of these sounds lower the body of the tongue, making this method unsuitable for transferring the bolus. Performing the "ee" maneuver allowed the bolus to easily move to the pharynx because the tongue was elevated at a higher position in the mouth. The front of the tongue is most elevated during "ee" vocalization compared to the other vowels [7,8]. Palatal lift prosthesis with a flexible lift (f-PLP) can improve the oral to pharynx bolus transportation by relieving the blockage at the fauces [9]; however, the "ee" maneuver requires no specific tools or preparation. Combined with a reclining posture, gravity facilitates the flow of the bolus from the oral cavity into the pharynx [10]. The bolus flows from the oral cavity into the pharynx during exhalation. Therefore, this maneuver has a low risk of aspiration. However, VF should be performed to evaluate the risk of aspiration and indications. When clinicians introduce this maneuver in a clinical setting, monitoring of clinical signs (i.e., respiratory status and choking) and percutaneous oxygen saturation are recommended.

The "ee" maneuver may be one of the techniques that can be used to proceed with direct therapy with food. This technique may be a good indication for patients with oral stage dysphagia due to pseudobulbar palsy blocking at the fauces caused by contact of the front of the tongue with the palate. The blockage should be confirmed in the lateral view of the VF. Furthermore, the cognitive function should be preserved to follow simple vocalization instructions. If the cognitive function is severely impaired and the patient cannot follow the vocal instructions, this technique would not be applicable. However, this maneuver might be effective in patients who have indications. Patients with dysphagia like the present case are not common in clinical settings. However, if the indications for this maneuver are met, this method may be effective.

This study has several limitations. First, this was a single case report; hence, future evaluation of additional cases is necessary. In the authors' clinical experience, the "ee" maneuver has been successful in other dysphagia patients with pseudobulbar palsy, but the effectiveness of this maneuver needs to be studied. Second, the pathology behind dysphagia was limited to pseudobulbar palsy. Additional studies are necessary to determine whether this maneuver can be successfully used for other types of dysphagia.

## Conclusions

In summary, the "ee" maneuver can be used as a swallowing maneuver to improve bolus transport from the oral cavity to the pharynx in patients with pseudobulbar palsy. Further studies are needed to evaluate the effectiveness of this maneuver.
